# An Unusual Helix Turn Helix Motif in the Catalytic Core of HIV-1 Integrase Binds Viral DNA and LEDGF

**DOI:** 10.1371/journal.pone.0004081

**Published:** 2009-01-01

**Authors:** Hayate Merad, Horea Porumb, Loussiné Zargarian, Brigitte René, Zeina Hobaika, Richard G. Maroun, Olivier Mauffret, Serge Fermandjian

**Affiliations:** 1 LBPA, CNRS (UMR 8113)–Ecole Normale Supérieure de Cachan, Cachan, France; 2 Département des Sciences de la Vie et de la Terre, Faculté des Sciences, Université Saint Joseph, CST-Mar Roukos, B. P. 1514, Beyrouth, Liban; Monash University, Australia

## Abstract

**Background:**

Integrase (IN) of the type 1 human immunodeficiency virus (HIV-1) catalyzes the integration of viral DNA into host cellular DNA. We identified a bi-helix motif (residues 149–186) in the crystal structure of the catalytic core (CC) of the IN-Phe185Lys variant that consists of the α_4_ and α_5_ helices connected by a 3 to 5-residue turn. The motif is embedded in a large array of interactions that stabilize the monomer and the dimer.

**Principal Findings:**

We describe the conformational and binding properties of the corresponding synthetic peptide. This displays features of the protein motif structure thanks to the mutual intramolecular interactions of the α_4_ and α_5_ helices that maintain the fold. The main properties are the binding to: 1- the processing-attachment site at the LTR (long terminal repeat) ends of virus DNA with a K_d_ (dissociation constant) in the sub-micromolar range; 2- the whole IN enzyme; and 3- the IN binding domain (IBD) but not the IBD-Asp366Asn variant of LEDGF (lens epidermal derived growth factor) lacking the essential Asp366 residue. In our motif, in contrast to the conventional HTH (helix-turn-helix), it is the N terminal helix (α_4_) which has the role of DNA recognition helix, while the C terminal helix (α_5_) would rather contribute to the motif stabilization by interactions with the α_4_ helix.

**Conclusion:**

The motif, termed HTHi (i, for inverted) emerges as a central piece of the IN structure and function. It could therefore represent an attractive target in the search for inhibitors working at the DNA-IN, IN-IN and IN-LEDGF interfaces.

## Introduction

The integration of the HIV-1 genome into the host cell chromosome is mediated by the viral integrase (IN) [1]–[6]. The enzyme catalyzes a multi-step reaction i.e., 3′-end processing and strand transfer, to integrate a linear DNA copy (cDNA) of the retroviral genome into the host cell DNA [2], [7], [8]. The retroviral DNA integration mimics that of insertion elements and bacteriophage Mu transposons [9]–[11] and bears resemblance to the RAG1/2 recombinase [12].

**Figure 1:**
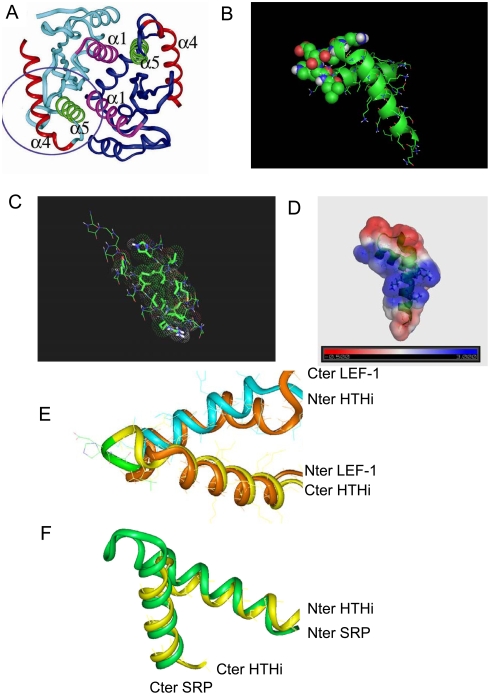
Identification of an “inverted” HTH motif (HTHi) at the catalytic core surface of integrase (PDB ID 1BIU [20]). a). Crystal structure of the catalytic core domain, associated into a dimer. b). Representation of the HTHi motif, with the loop residues shown by van der Waals spheres. c). The side chain residues involved in intramolecular contacts, shown by sticks and van der Waals spheres. d). The electrostatic potential at the solvent-accessible surface; the Lys-156, Lys-159 and Lys-160 residues are shown by sticks. e). HTHi motif of IN, superimposed onto the “classical” HTH motif of the HMG (highly mobile group) protein LEF-1 (lymphoid enhancer binding factor, PDB ID 2LEF, brown). f). HTHi motif of IN, superimposed onto the HTHi motif of the Signal Recognition Particle (PDB ID 2FFH, green).

The HIV-1 IN is essential for the viral life cycle and is therefore an attractive target for developing anti-HIV drugs [13], [14]. The enzyme (288 amino acid residues, 32 kDa) has three well defined structural domains: an N terminal domain (residues 1 to 49), a central catalytic domain or catalytic core, CC (residues 50 to 212), and a C terminal domain (residues 213 to 288) [15]–[17]. Several crystal structures of the CC domain and of two-domain fragments (CC domain linked either to the C terminal domain or the N terminal domain) have been already resolved by X-ray crystallography [18]–[25] while the N terminal and C terminal domains have been analyzed in solution by NMR [26], [27]. Each domain, taken separately, forms a dimer and this is true also true for the N terminal-CC and the C terminal CC bi-domains [18]–[29]. The CC dimer (Fig. 1a) is organized around a two fold axis with a large interface involving, in particular, helices α_1_ and α_5_ (residues 172–184) [18], [30]. Other retroviral IN CC structures display the same dimer boundary, indicating that this type of interface is biologically relevant.

Actually, cross-linked dimers have been shown to be active for 3′-processing and single end integration [31]. Yet, a large number of data suggest that the tetramer is the form stabilizing the synaptic complexes of IN with the two viral DNA ends and appears to be the form required for the strand transfer [32]–[37]. Several theoretical models of the DNA-IN complexes have proven the relevance of tetramers to position the viral and cellular DNA partners at reactive distance [38]–[41].

The CC domain is organized in five β-strands surrounded by six α helices (α_1_ to α_6_), and contains a highly conserved catalytic D, DX_35_E motif embedded in a protein RNase H fold [17], [20], [21]. The amphipathic α_4_ helix, (residues 148–167), which protrudes at the protein surface, bears the catalytic residue Glu-152 and several other residues, such as Gln-148, Lys-156 and Lys-159, which have been shown to be important for the binding of IN to DNA and for virus survival. In the crystal structure of CC bound to the inhibitor 5CITEP (1-(5-chloroindol-3-yl)-3-(tetrazoyl)-1, 3-propanedione enol) among the six protein-drug interactions, five involve amino acid side chains of the α_4_ helix [42], confirming the relevance of the α_4_ helix to IN function [41], [43]–[47].

The propensity of IN to associate and form aggregates is the main barrier to the study of its structure and interactions by physical-chemical means including the widely used x-rays and NMR techniques. To date the only detailed depiction of the IN-DNA complex is provided by molecular modeling [38]–[41]. To overcome the difficulties inherent to the use of the entire enzyme we decided to dissect its properties and to analyze it part by part. Our laboratory has previously shown, in line with a systematic search by Li et al. [48], that peptides deriving from the α_4_ helix had inhibitory activity against IN [49], [50]. This was also the case for peptides reproducing the sequences of the α_1_ and α_5_ helices involved in the CC dimer interface (α_1_∶α_5′_ and α_1′_∶α_5_) [30]. We have also shown that the peptide region 163–171, encompassing both the turn and the N and C terminal parts of the α_4_ and α_5_ helices, respectively, was a strong epitope and antibodies refined against this region inhibited both the 3′ processing and strand transfer reactions in *in vitro* assays [49]. Interestingly, the epitope region, in particular the residue Gln168 important for turn integrity, IN dimer formation and virus replication, has been shown to share hydrogen bonds with the IBD (IN binding domain) of LEDGF/p75 (lens epidermal derived growth factor), a transcriptional coactivator that is also an essential HIV integration cofactor *in vivo* [49], [51]–[56]. Moreover, it has been assumed that the peptide region 161–173 superposing our epitope was involved in the nuclear import of IN [57], since a peptide reproducing this sequence caused the active nuclear import of BSA tethered to it [57], [58]; the matter was somehow reassessed in so far as the fact that Val-165 and Arg-166 did not play the anticipated specific roles in the nuclear localization of HIV-1 pre-integration complex [59], [60].

Actually, the α_4_ and α_5_ helices form a stable bi-helix fold at the protein surface of the CC crystal structure (Fig.1a–f). This recalls the well-known HTH (helix-turn-helix) motif of proteins specialized in DNA recognition [61]. Such motifs are generally associated with biological functions, protein structures and even evolutionary history. To assess the possible role of this bi-helix in IN we carried out an analysis of the structural and binding properties of the corresponding synthetic peptide (residues Gly-149 to Lys-186) using circular dichroism (CD) [43] and fluorescence. The peptide dealt with in this work represents the sequence found in the IN-CC Phe185Lys variant (Table 1a). It has been logically preferred to the native wt sequence (Table 1a) because it is the Phe185Lys variant that was used in the various crystallizations [20] and co-crystallizations so far reported [42], [52]. The Phe185Lys mutation is not expected to affect the interactions with LTR and LEDGF IBD [51], [52]. We have nevertheless performed a number of experiments (not shown here) to verify that the structure of the wt bi-helix was similar to that of the mutant.

**Table 1:** Sequences of Peptides and Oligonucleotides.

Peptides*	wt HTHi	SQG_149_VIESMNK_156_ELKKIIGQVRDQ_168_ AEHLKTAVQMAV**W**IHNF_185_K
	HTHi	**Y**G_149_VIESMNK_156_ELKKIIGQVRDQ_168_ AEHLKTAVQMAVFIHN**K**_185_K**A**
	α_4_	SQG_149_VVESMNK_156_ELKKIIGQVR**Y**
	K156	SQ**A**_149_ **KL**E**E**MNK_156_ELKK**LLA**QVR**A**Q_168_ **W**
	INH5	DQ_168_ AEHLKTAVQMAVFIHN**Y**_185_K**A**
**Oligonucleotides** **	LTR34	_5_′-ACTGCTAGAGATTTTCC**TTT**GGAAAATCTCTAGCAGT-_3′_
	LTR34f5′	_5_′-**_f_**ACTGCTAGAGATTTTCC**TTT**GGAAAATCTCTAGCAGT-_3′_
	LTR34fm	_5_′-ACTGCTAGAGATTTTCC**TFT**GGAAAATCTCTAGCAGT-_3′_
	CRE	_5_′-**_f_**GAGATGACGTCATCTC-_3′_

* The HTHi peptide reproduces the Gly-149 to Lys-186 sequence of HIV-1 IN Phe185Lys [20]. The loop region is underlined. The mutated residues are in bold characters. An N-terminal labeled carboxyfluorescein derivative of HTHi was also used (not shown). The peptides α_4_, K156 and INH5 were dealt with in [30], [43], [50].

** The LTR34 oligonucleotides are designed to adopt a hairpin conformation, as already reported [43]. The hairpins contain a 17 bp stem, reproducing the HIV-1 U5LTR end, and a loop formed by three thymine nucleotides (TTT in bold characters). The fluorescein reporter group, f, is grafted either to the 5′ extremity (LTR34f5′, CRE) or to the central T residue (F) of the loop (LTR34fm).

Generally, the HTH motifs are strongly influenced by their context within the proteins and often lose their original biological functions when they are isolated from their protein environment. Here, we show that the isolated HTH motif continues to fulfill three of the major functions of the whole parental enzyme: i) it recognizes the U5LTR extremity of viral DNA at the attachment-processing site in a specific manner i.e., it preserves its viral DNA binding property; ii) it self-associates and associates to IN i.e., it reproduces some of the enzyme oligomerisation behavior, and iii) it binds to the IBD of LEDGF but not to its Asp366Asn-IBD variant lacking the essential Asp366 residue i.e., it maintains its ability to interact with specific partners.

## Results

### The HTHi conformation

The bi-helix motif we focus on (α_4_ helix-turn-α_5_ helix) occurs in the peptide segment from Gly-149 to Lys-186 of the IN-Phe185Lys variant. Like many classical HTH motifs, this bi-helix is exposed at the protein surface where it is largely accessible to solvent, proteins, DNA and organic ligands (Fig. 1a) [20]. The two helices are linked by a flexible turn of 3 to 5 residues bringing the amphipathic α_4_ helix (situated in N terminal position) onto the C terminal α_5_ helix through an angle of about 120°(Fig. 1b). The helices interact with each other through 16 mainly hydrophobic side chain–side chain contacts (Fig. 1c and Table 2). The juxtaposition of the helices generates a positive electrostatic potential exposed at the solvent accessible surface (Fig. 1d). The only difference between the present motif and the standard HTH motif is the inversion of position of the DNA recognition helix (α_4_ helix) and of the so-called stabilizing helix (α5) (Fig. 1e). Actually, the bi-helix motif of IN resembles the RNA-recognizing motif of the SRP (Signal Recognition Particle, Fig. 1f), where the role of the helices are inverted, the N terminal one recognizing the DNA and the C-terminal one stabilizing the structure [62]. The bi-helix motif was therefore hereafter called HTHi (helix-turn-helix inverted).

**Table 2:** Main interactions within HTHi (helices α_4_ and α_5_) and of HTHi with the neighbouring β-sheet in the IN catalytic core (data from PDB ID 1BIU, [20]).

		α_4_-helix											β-sheet			
		V150	I151	M154	N155	E157	L158	I161	I162	V165	R166	Q168	Y83	I84	A86	V88
	L172								**Φ**		**Φ**					
	A17_5_								**Φ**		**Φ**					
	V176								**Φ**						**Φ**	**Φ**
	Q177														**H**	
	M178									**Φ**		**Φ**				
**α_5_**-**helix**	A179						**Φ**	**Φ**	**Φ**							
	V180						**Φ**								**Φ**	
	I182							**Φ**		**Φ**						
	H183					**E**		**Φ**								
	N184												**Φ**	**Φ**		
	I186					**E**										
	Q62	**Φ**	**Φ**													
	N64		**Φ**		**E/H**											
**β**-**sheet**	L68								**Φ**							
	I73								**Φ**							
	V75				**Φ**		**Φ**									
	I84			**Φ**			**Φ**									

φ: hydrophobic contacts; H: hydrogen bonds; E: Electrostatic interactions.

**Figure 2:**
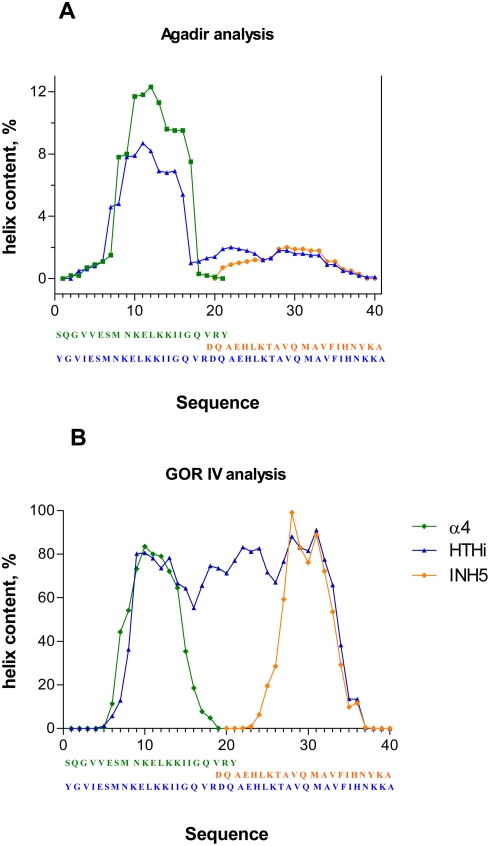
Secondary structure predictions. Results provided by: a). Agadir [64] and b). GOR IV [63] for the helical propensities of HTHi (blue), α_4_ (green) and INH5 (orange). The predictions with Agadir were performed at pH 7, at a temperature of 25°C.

The comparison of the GOR IV [63] and AGADIR [64] predictions strongly suggest that long distance interactions–like those stabilizing the protein tertiary structures–are needed besides the short distance interactions in order to keep the helix secondary structure in the HTHi motif (Fig. 2). Examination of the reported crystal structures shows that long range interactions occur in great number inside the HTHi motif itself i.e., between the α_4_ and the α_5_ helices (there are 16 such interactions, listed in Table 2), but also between the HTHi motif and other components of the protein (for example, there are 16 interactions between HTHi and the neighboring β-sheet–Table 2). Accordingly, a loss of helicity could be feared upon isolation of the motif from the protein context.

**Figure 3:**
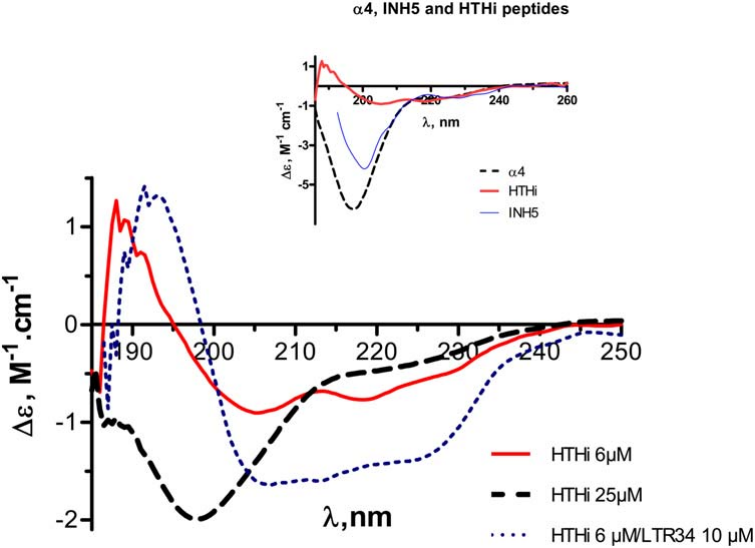
Circular dichroism. Spectra of peptide HTHi at 6 µM (^__^, red) and at 25 µM (—, black), and difference spectrum of the complex [HTHi (6 µM)+LTR34 (10 µM)] minus the CD spectrum of LTR34 (10 µM) (……‥, blue). Insert: CD spectrum of HTHi compared to the spectra of its component peptides, α_4_ (after Figure 6 in [43]) and INH5 (after Figure 3b in [30]). Recall that INH5 comprises both the α_5_ helix and the turn linking α_5_ to α_4_ (Table 1a).

CD analysis indicates that the secondary structure of the HTHi peptide in solution is strongly concentration dependent. At 6 µM, with its two negative bands at 208 nm and 222 nm, the CD spectrum of HTHi reflects a helical content of 10–15% (full line curve in Fig. 3). Upon increasing the peptide concentration to 25 µM, the CD spectrum manifests an important change. This is illustrated by the reduction of the broad negative band at 222 nm, together with the replacement of the negative band at 208 nm by a larger band mostly centered at 198 nm that bears a component with positive tendency at 190 nm, signaling the presence of a weak amount of helix (dotted line curve in Fig. 3).

The above CD experiments reveal the propensity of HTHi to form oligomers in solution at high concentration. It appears that the setting up of intermolecular interactions occurs at the detriment of the intramolecular interactions between the α_4_ and the α_5_ helices and impairs the stability of the HTHi fold and consequently that of the of the helix secondary structure. We argue that the weakening of these intramolecular interactions is the main cause leading to a decrease of the helix content of the motif at high peptide concentration.

The stabilization of the HTHi fold by the intramolecular interactions between the α_4_ and α_5_ helices is highlighted by the comparison of the CD spectrum of HTHi with the spectra of its component peptides, α_4_ and INH5 (inset to Figure 3). The fact that the helix content of HTHi is greater than that of peptides α_4_ [43] and INH5 [30] is particularly obvious in the low-wavelength region of the CD spectra. Indeed, while the separate components of the bi-helix show important negative bands in the 195 nm domain of the spectrum, this feature vanishes in the spectrum of HTHi, which develops a positive band below 190 nm, compatible with a helical structure.

### Binding of the HTHi motif to viral DNA

We carried out CD and fluorescence experiments to demonstrate that peptide HTHi binds with a good affinity to the viral LTR DNA.

To begin with, CD was used to assess the conformational changes undergone by the HTHi peptide upon interacting with LTR34 DNA (Table 1). Indeed, the latter exerts a helix stabilizing effect that is attested in the difference spectrum (i.e., peptide-DNA complex minus DNA) by the increase of the positive band at 190 nm and of the two negative bands at 208 and 222 nm, less perceptible in the spectra of unbound HTHi (Fig. 3). The HTHi motif practically doubles its helical content when binding to LTR34. This behavior is not unique: we have already shown that a peptide derived from the b-ZIP (basic-zipper) motif of the c-jun protein manifested the same helix stabilization upon binding to DNA [65].

**Figure 4:**
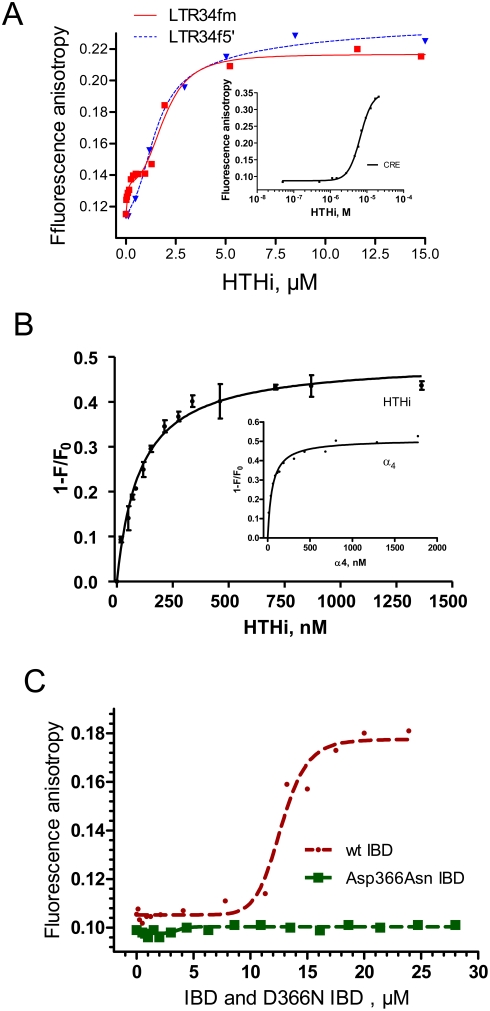
Fluorescence. a). Interaction with DNA. Fluorescence anisotropy titration by peptide HTHi of LTR34fm (squares, red) and LTR34f5′ (triangles, blue). Inset: titration of CRE by peptide HTHi. In all cases, the fluorescein-labeled DNA was at 20 nM. The K_d_ values obtained by curve fitting (with ±10% uncertainty) are: 0.05 µM and 1.9 µM for LTR34fm; 2.9 µM for LTR34f5′; and 6.6 µM for CRE. b). Interaction with wt IN. Quenching of the intrinsic fluorescence of wt IN by peptide HTHi. Inset: Titration of wt IN by peptide α_4_. In all cases, the wt IN monomer concentration was 100 nM. The midpoints of the titration curves are at 104±8 nM and 53±5 nM for peptides HTHi and α_4_ respectively. c). Interaction with LEDGF-IBD. Fluorescence anisotropy titration of fluorescein-grafted HTHi peptide (50 nM) by LEDGF-IBD (wt IBD, round symbols, and mutated IBD, squares). The K_d_ of the wt IBD-HTHi interaction is 12.3±0.3 µM, with a Hill number of 11±3.

The binding parameters were determined by fluorescence anisotropy measurements. A representative binding isotherm illustrating the titration of the oligonucleotide LTR34fm (Table 1b), by peptide HTHi is shown in Fig. 4a. The binding curve is biphasic, whereas that obtained with the structural analog LTR34f5′ is monophasic. With LTR34fm, each plateau indicates the saturation of an interaction. The first, corresponding to the high affinity binding, has been assigned to the specific recognition by HTHi of the extremity of viral DNA [43]. The high affinity is abolished by the grafting of the fluorescein moiety at the 5′extremity (LTR34f5′) (Fig. 4a). The dissociation constants of peptide HTHi, determined by curve fitting, are K_d1_ = 0.05 µM and K_d2_ = 1.9 µM for LTR34fm and K_d_ = 2.9 µM for LTR34f5′ (Fig. 4a). Note that the non-specific binding to the negative control sequence (CRE, cAMP response element) is within this latter range of affinities (K_d_ = 6.6 µM).

### Interaction of the HTHi motif with wt IN

In a previous study, based on the intrinsic fluorescence properties of IN conferred by its six Trp residues, we have shown the ability of peptide INH5 (Table 1a) to interact with IN with a concentration midpoint (C_0.5_) of 168 nM [30]. The interaction, occurring at the α_1′_∶α_5_/α_1_∶α_5′_ interface, caused the dissociation of the tetramers and dimers of IN [30]. The experiments performed with HTHi under conditions similar to those we had previously used for peptide INH5 [30], show that HTHi interacts with wt IN (at 100 nM monomer concentration) with a C_0.5_ value of 104 nM, while the peptide α_4_, taken alone, binds to wt IN with a C_0.5_ value of 53 nM (Fig. 3b). Indeed, we had previously shown that peptide α_4_ interacted with IN likely by forming a coiled-coil structure with its counterpart in the protein [50], [66].

### Interaction of the HTHi motif with LEDGF

Fluorescence anisotropy was also used to asses the binding of the HTHi motif to IBD-LEDGF. A fluorescein-labeled HTHi peptide (Fig. 4c) was prepared for this purpose and titrated by both the wt and the Asp366Asn mutated LEDGF IBD protein ligands. It has been previously shown that Asp366 is essential for the binding of LEDGF to IN [52], [54]. The overall pattern unambiguously shows that only the wt IBD binds HTHi. Curve analysis provides a K_d_ of the order of 12 µM. We find that HTHi reproduces the properties of the CC of IN in so far as the recognition of LEDGF is concerned. The essential role of the IBD residue Asp-366 in the interaction is confirmed.

## Discussion

The so-called HTHi motif (α4-turn-α5) identified at the IN protein surface presents an unexpected multiplicity of functions revealed by the study of the corresponding synthetic HTHi peptide. In the latter, hydrophobic contacts between its α_4_ and α_5_ arms confer stability to the ensemble, i.e., to the tertiary structure and the helical secondary structure. The α_4_ helix is more basic than the α_5_ helix, which is consistent with its DNA binding property. However, only a stable preformed scaffold permits the proper spatial orientation of the interactive amino acid side chains and their adjustment onto their complementary nucleotides of the DNA target without large conformational change and therefore large consumption of energy. This improves the affinity and confers specificity to the binding.

Our results strongly suggest that helix stability in the HTHi motif is essential to the specific recognition of viral DNA. In particular, the α_4_ peptide taken in isolation, which does not benefit of the HTHi stabilizing context, is a very poor DNA binder. The difference of DNA binding affinity between the peptides HTHi and α_4_ can be attributed to the larger conformational change that the latter must undergo in order to adopt the bound helical conformation.

In a previous work we pointed out the importance of the conformational entropy in the binding of the α_4_ helix to the viral DNA. In order to reduce this effect, we had designed an analog of the α_4_ peptide that had its secondary structure stabilized by helicogenic mutations (Gly149Ala, Ile161Leu, Ile162Leu and Gly163Ala). The resulting peptide, K156, was a good mimic of the α_4_ helix in the protein context and was better organized for binding than peptide α_4_. The use of peptide K156 with the oligonucleotide LTR34fm permitted the identification of a high affinity site (K_d1_ = 2.1 nM), corresponding to the attachment site of the enzyme on the six outermost bases of viral DNA and of a low affinity site in the micromolar range (K_d2_ = 54 µM) corresponding to the non-specific binding of IN[43]. The grafting of the bulky fluorescein at the 5′ extremity (LTR34f5; Table 1b), i.e., at a position in close proximity to the 3′-processing site, suppressed the high affinity binding mode of K156 but preserved the low affinity mode. The absence of effect on the binding to the low affinity site from the grafted fluorescein suggested that this site was distant on the DNA from the high affinity site [43].

The behavior of peptide HTHi is similar to that of peptide K156. With HTHi there are two binding modes as well, one with a low dissociation constant in the nanomolar range ( K_d1_ = 50 nM) and the other with a high dissociation constant in the micromolar range ( K_d2_ = 1.9 µM ), in the case of LTR34fm; and there is only one binding mode in the micromolecular range (K_d_ = 2.9 µM ) in the case of LTR34f5′ (Fig. 4a). The non-specific binding of peptide HTHi to the negative control sequence, CRE, is within this latter range of affinities; K_d_ = 6.6 µM. ; comparatively, that of peptide K156 was 16 µM [43].

Thus, the two peptide ligands, HTHi and K156, present the same DNA binding properties. The two binding modes, of which the first appears specific for the intact processing-attachment site of LTR DNA, are present in the two cases, albeit with different affinities. The role of conformation is noteworthy. Peptide K156, whose secondary structure has largely benefited from helicogenic mutations, manifests a greater affinity for viral DNA than peptide HTHi, in which the α_4_ helix component, certainly stabilized by its interactions with the α_5_ helix, is nevertheless less stable than peptide K156. This is consistent with the idea of a higher pre-formed conformation for binding of peptide K156 compared with peptide HTHi that lowers the entropy cost of interaction of the former. As a corollary, the isolated peptide α_4_, which mostly displays random conformation because of the absence of either helicogenic mutations in its sequence or helix stabilization conferred by the interaction with α_5_, is completely unable to specifically bind the viral DNA.

X-ray crystallographic studies of the co-crystal of CC with 5CITEP also illustrate the importance of the HTHi α_4_ helix in the IN enzyme. Results strongly suggest that the a4 helix would be the primary target of the DKA (diketo acid/aryl) family of inhibitors of IN acting on the 3′ processing step [14], [42], [67]. In the complex, the drug interacts with five residues of the α_4_ helix and at least four of them (Lys-156, Lys-159, Gln-148 and Glu-152) are also implicated in the stabilization of the viral DNA-CC complex [14], [43].

Concerning the role of the α_5_ helix, it is clear from the various crystal structures that it participates to both the dimerization of the enzyme and the stabilization of the motif [18]–[29]. There are examples in the literature showing that the so-called stabilizing helix of HTH motifs is involved in the protein dimerization. This is the case of topoisomerase II, in which the binding site to DNA is fashioned from the interaction of two HTH motifs [68]. The implication of the α_5_ helix in the enzyme structure and activity has been highlighted in our previous report showing that peptide INH5, which comprises the α_5_ helix, inhibits IN by dissociating the IN dimers and tetramers [30].

The role of the HTHi motif is not limited to that of its α_4_ and α_5_ helix components. At the solvent accessible surface, in the center of the motif, the electrostatic potential is uniform and positive and it changes sign at the turn connecting the α_4_ and α_5_ helices (Fig. 1d). The 161–173 region is a strong epitope and also a protein binder [49], [51]–[56]. Antibodies enriched against this epitope inhibit both the 3′ processing and the strand transfer reaction [49]. The particular variation of the electrostatic potential at the solvent accessible surface in that region is compatible with these functions. As a matter of fact, a peptide corresponding to the epitope region has been shown to mediate the nuclear import of the covalently linked serum albumin [58], although this region has been incorrectly proposed as being a possible NLS (nuclear localization signal) [57]. Nevertheless, the present results confirm the propensity of the epitope region to associate with important proteins, such as LEDGF. The co-crystal structure of CC bound to LEDGF-IBD shows that the epitope region is recognized by LEDGF. The formation of a complex between IN and LEDGF is biologically relevant and is a prerequisite to the strand transfer into the cell chromosome [51]–[56]. In the complex, there are numerous stabilizing contacts, such as those involving the α_3_ helix and a portion of the α_1_ helix, but it is the turn region of HTHi ( residues Glu-170, His-171 and Gln-168 ) that makes the major contribution, particularly in binding the hotspot residue Asp-366 and the residue Ile-365 of LEDGF[51], [52], [54]. Our results are compatible with these findings. Resembling the IN CC, the peptide HTHi binds wt IBD, but not its Asp366Asn variant. We are thus allowed to conclude that the pattern of interactions found in the CC-IBD crystal is still present in the peptide HTHi-IBD complex.

All together, the results mentioned above indicate that the HTHi motif identified in IN behaves like a multifunctional entity. Being involved in enzyme oligomerisation (via its α_5_ helix), in LTR end recognition (via its α_4_ helix) and in binding to LEDGF (via the turn region, which also acts as a strong epitope), it emerges as a central piece of both the IN structure and activity. Moreover, the α_4_ helix of HTHi could be the target of inhibitors belonging to the DKA family, as suggested by the crystal structure of 5 CITEP bound to IN CC [14], [42], [67]. We propose that HTHi could constitute a reliable model for the study of new inhibitors acting at the IN-IN, the IN-DNA and the IN-LEDGF interfaces. This issue will be further addressed possibly by looking for peptidomimetics to the α_4_ and α_5_ helices, as well as to the epitope region–with the aim of transforming them into therapeutic agents.

## Materials and Methods

### Peptides

We used the synthetic peptides shown in Table 1a. They were made according to the Fmoc procedure, as already reported for peptide α_4_, for its “stabilized” analogue i.e., peptide K156, and for the peptide INH5, derived from the α_5_ helix [30], [43]. Peptide α_4_ reproduces the α_4_ helix sequence of IN; peptide K156 is a variant of the α_4_ peptide resulting from the replacement of weakly helicogenic residues by more helicogenic ones in several biologically irrelevant positions [43]; peptide INH5 contains both the α_5_ helix and the turn connecting the α_5_ helix to the α_4_ helix [30]. The peptides HTHi and wt HTHi reproduce the sequences of the HTHi motifs in the IN-Phe185Lys variant and in wt IN respectively. A version of the HTHi peptide with a carboxyfluorescein at the N-terminus has also been prepared. Tyr or Trp aromatic residues, when absent in the native sequences, were purposely added in order to enable peptide quantification by UV absorption spectra. Peptide concentrations were determined using a molar absorption coefficient at 280 nm equal to 1 280 M^−1^ cm^−1^ for the tyrosine-containing peptides α_4_, INH5 and HTHi and 5600 M^−1^ cm^−1^ for the tryptophane-containing peptides K156 and wt IN.

### DNA oligonucleotides

The oligonucleotides (Table 1b) were purchased from Cybergene ESGS (France) and Eurogentec (Belgium). The choice of monomolecular hairpin-forming oligonucleotides, rather than bimolecular duplex-forming ones, was motivated by the need for stability under the low concentrations inherent to the fluorescence anisotropy experiments. The fluorescein reporter group is grafted either to the central T nucleotide (LTR34fm) or to the 5′ extremity (LTR34f5′ and CRE). The CRE (cAMP responsive element) sequence was used as negative control. The version of LTR34 without fluorescein was used in CD studies.

### Proteins

Fluorescence titrations were performed with the wild-type IN (wt IN) [69]. The presence of Trp in the enzyme (IN contains six Trp residues) but not in the peptides, was exploited when performing intrinsic fluorescence quenching titrations of the former. The wt IBD is the fragment 347–471 of LEDGF (GST removed) [33], [51], [52]. The mutant IBD is the Asp366Asn version of the former. Like the whole protein, these contain no Trp. The concentrations of wt IN (33.781 kDa) and of the IBD fragments (16.542 kDa) were estimated from UV absorption at 280 nm, using the molar absorptivities of 46542 M^−1^ cm^−1^ and 1400 M^−1^ cm^−1^ respectively. The proteins were used in the “reaction buffer” containing 20 mM HEPES (pH 6.8), 10 mM MgCl_2_ and 10 mM DTT.

### Secondary structure predictions

Secondary structure predictions were carried out using the AGADIR and GOR IV computer programs available on the web respectively at http://www.embl-heidelberg.de/serices/serrona/agadir-start.html and http://pbil.univ-lyon1.fr/ (Pôle Bioinformatique Lyonnais, France) [63], [64]. The AGADIR prediction considers short range interactions between residues and provides helical propensity per residue of peptides in solution, independently of tertiary structure interactions, with the possibility of selecting the pH and the temperature conditions. GOR IV uses parameters derived from crystallographic data of proteins. It thus provides more realistic structure predictions for peptide segments submitted to tertiary and quaternary structures constraints i.e., within the protein environment. Thus the two types of predictions can be used in conjunction to find the impact of the protein context on the secondary structure of its peptide elements, to identify the effect of mutations on secondary structures and to select the mutations that would reinforce the helicity of peptides, as was done in the case of peptide K156 (Table 1a) [43].

### CD Spectroscopy

CD spectra were recorded on a Jobin-Yvon CD6 dichrograph (HORIBA Jobin-Yvon, France). Peptide concentrations varied from 5 to 25 µM in the “assay buffer”, containing 40 mM sodium phosphate, 0.5 mM EDTA, pH 6.0. The samples, incubated 10 min at the chosen temperature to allow the solutions to reach their equilibrium state, were placed in thermally jacketed cells with a 1 mm path length. The spectra, recorded in 1 nm steps, were averaged over ten scans and corrected for the base line, then presented as differential molar absorptivities per residue, Δε (M^−1^ cm^−1^), for peptides, and as difference spectra (complex minus DNA), for peptide-DNA complexes. For the HTHi-LTR interaction, aliquots of peptide were added to LTR34 (10 µM) and the control spectrum of unliganded LTR was subtracted from that of the complex. Recall that in a simple random coil – α helix equilibrium, the α-helix content of peptides could be approximated by the relation: P_α_ = −[Δε_222_×10] (P_α_: percentage of α helix; Δε_222_: differential molar absorptivity per residue at 222 nm) [70].

### Modeling

We used the Insight II® program (Accelrys Software Inc., CA, USA) to estimate distances between the various atoms in HTHi and between HTHi and the β-stands located in the proximity, using the CC IN crystal structure (PDB ID 1BIU) [20] (Fig. 1 and Table 2). Graphics was by PyMol (http://www.pymol.org) [71] with the APBS software (http://apbs.sourceforge.net/) [72].

### Fluorescence Measurements

Intrinsic fluorescence quantum yield and fluorescence anisotropy studies were carried out with a Jobin-Yvon Fluoromax II instrument (HORIBA Jobin-Yvon, France) equipped with an ozone-free 150 W xenon lamp. The samples (800 µl) were placed at 5°C in thermally jacketed 1 cm×0.5 cm quartz cells. At least ten measurements for each titration point were recorded with an integration time of 5 sec.

*The intrinsic fluorescence* [73] of wt IN was measured at 100 nM monomer concentration, in the “reaction buffer” (see above). The Trp residues were excited at 295 nm and emission was recorded between 305 and 450 nm, with 2 nm and 5 nm excitation and emission slit widths, respectively. A maximal emission was obtained at 337 nm. Quenching of the fluorescence of IN following the addition of HTHi or of peptide α_4_ (devoid of Trp) was expressed as 1-F/F_0_, where F_0_ is the fluorescence of the enzyme in the absence of peptide.

*Fluorescence anisotropy titration* [74], [75]. Fluorescein-labeled oligonucleotides were used at 20 nM in the “assay buffer” (see Table 1b and above). With fluorescein as fluorophore, excitation and emission were at 488 nm and 516 nm, with 4 nm and 5 nm slits, respectively. Peptides were added to DNA as successive aliquots. For each anisotropy measurement, the parallel 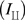 and the perpendicular 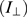 intensities of the background solution (i.e., buffer and protein contributions) were subtracted from those of the sample.

The fluorescence anisotropy of the fluorescein-conjugated version of HTHi (50 nM) titrated by wt and mutated IBD in the “assay buffer” was used to evaluate the K_d_ of the LEDGF-HTHi complex.
